# Indications for diagnostic use of nuclear medicine in rheumatology: A mini-review

**DOI:** 10.3389/fmed.2022.1026060

**Published:** 2022-09-28

**Authors:** Martin Wenger, Michael Schirmer

**Affiliations:** ^1^Department of Nuclear Medicine, Klinikum Bad Hersfeld, Bad Hersfeld, Germany; ^2^Clinic II, Department of Internal Medicine, Medical University of Innsbruck, Innsbruck, Austria

**Keywords:** imaging, scintigraphy, tomography, magnetic resonance, fluorodeoxyglucose, Technetium-99m, fluoride, giant cell arteritis

## Abstract

Nuclear medicine techniques allow important insights not only into oncologic, neurologic, and infectious conditions, but also for the assessment of rheumatic diseases. This review provides a brief, update on the potential role of nuclear imaging in rheumatology, especially on ^18^F-fluorodeoxyglucose (FDG) positron emission tomography for the diagnosis of giant cell arteritis and other large vessel arteritis according to international recommendations. Besides, the potential role of this and other nuclear imaging techniques for the rheumatologic practice are summarized. With ^18^F-fluoride as tracer for positron emission tomography, a new option for bone scintigraphy comes up, whereas the use of a semiquantitative sialoscintigraphy is no more supported for classification of Sjögren's syndrome according to current recommendations. Other techniques are used for different organ manifestations in systemic rheumatic diseases like for myocardial infarction and apoplectic insult.

## Introduction

The application of nuclear medicine techniques allows diagnostic insights not only into most oncologic, neurologic and infectious conditions, but also into selected rheumatic diseases. In rheumatology, ^18^F-fluorodeoxyglucose (FDG)-positron emission tomography (PET) can be considered as the nuclear medicine techniques most often requested by rheumatologists. According to a recent survey for diagnostic purposes, large vessel vasculitis, fever or increased erythrocyte sedimentation rate of unknown origin are the most common indications to request FDG-PET, while sarcoidosis, immunoglobulin G4-related disease, total joint replacement, constitutional symptoms, suspicion of malignancy, polymyalgia rheumatica (PMR) and suspicion of osteomyelitis are rare indications (1). Bone scintigraphy, and semiquantitative sialoscintigraphy have been established in rheumatology, but today are less frequently needed in rheumatology as compared to orthopedics and oncology, respectively.

The combined use of nuclear medicine techniques together with computerized topography (CT) or magnetic resonance imaging (MRI) provides a combination of functional data of increased cellular metabolism together with exact localization and description of the affected anatomic structures. Amongst experts including 80% rheumatologists with ≥5 years of experience in FDG-PET, 95% already utilize PET–CT, 9% PET–MRI and only 12% standalone PET (respondents were allowed to indicate more than one option) (1). For the future, the increasing availability of total body imaging will certainly have a revolutionary impact on day-to-day practice of medicine (2).

This narrative mini-review summarizes the most important clinical aspects of nuclear medicine techniques—from the rheumatologist's and a nuclear medicine specialist's perspectives.

## Established nuclear medicine techniques for indications in rheumatology

### FDG-PET in large vessel vasculitis

#### General considerations

According to the 2012 Revised International Chapel Hill Consensus Conference Nomenclature of Vasculitides, inflammation of large vessels are typical for giant cell arteritis (GCA) and Takayasu arteritis (TAK), but may also present in variable vessel vasculitis like Behçet's disease and in isolated aortitis as single organ vasculitis (3). This consensus stated that “primarily because there are no specific biomarkers for TAK and GCA, it is not possible to know if any or all examples of single organ vasculitis aortitis are limited expressions of TAK or GCA.” This aspect has important implications on therapeutic decision-making, as controlled interventional studies are not available so far.

For the differentiation between isolated aortitis and TAK or GCA, imaging using FDG-PET with or without CT or MRI is helpful. Since about 20 years, FDG-PET has been introduced into clinical practice (4), and for diagnosis of non-cranial large vessel arteritis, first successful cases were reported back in 2003 (5), with more and more studies coming up in the following years. The most important milestones in the field were achieved in 2017 and in 2018 as outlined below.

#### FDG-PET for classification of giant cell arteritis in 2017

Since 1990, the American College of Rheumatology (ACR)-criteria GCA have been successfully applied (6). At this time, the focus on GCA was on temporal arteritis as the cranial form of GCA. Only later, the non-cranial forms of GCA were more and more realized, especially by using imaging tools for examining the aorta and its major branches.

In 2017, the diagnostic delay of non-cranial GCA was still reported with 17.6 weeks and thus supported the need of fast-track diagnostic pathways (7). In the same year, however, a large interventional trial was published reporting not only the effects of tocilizumab as an inhibitor of interleukin-6 receptor alpha in GCA, but also used PET as an imaging option for identifying patients with non-cranial GCA (8). In this trial, “diagnosis of GCA was based either on results of a temporal-artery biopsy showing features of GCA or on evidence of large- vessel vasculitis on angiography, CT or magnetic resonance angiography, or PET” (8).

Since then, FDG- PET/CT is accepted as an imaging technique to be included into considerations for both diagnosing and classifying GCA. Such use of imaging tools like the F-FDG-PET for the non-cranial forms of GCA without involvement of the temporal arteries had never been formally validated in combination with other parameters for their potential accuracy as classification tool. As this trial further led to the approval of tocilizumab for the indication of GCA by national and international authorities, the study can be considered both as a milestone for classification of GCA and introduction of a new treatment also for GCA in general including the non-cranial form of GCA (9).

#### 2018 recommendations for use of FDG-PET

In 2018, both a joint procedural recommendation of the European Association of Nuclear Medicine (EANM), the Society of Nuclear Medicine & Molecular Imaging (SNMMI) and the PET Interest Group (PIG) endorsed by the American Society of Nuclear Cardiology (ASNC), and a study group of the European Alliance of Associations For Rheumatology (EULAR) made recommendations for FDG-PET/CT imaging in large vessel vasculitis and polymyalgia rheumatica (PMR) (10, 11). These two recommendations are summarized in Table 1. Today, the use of FDG-PET/CT with or without angiography [FDG-PET/CT(A)] is well-established for diagnosing the non-cranial form of GCA, TAK and isolated aortitis. The consensus of experts in the field include the aspects of patient preparation, FDG-PET/CT(A) acquisition and interpretation for the diagnosis and follow-up of patients with suspected or diagnosed large vessel vasculitis and/or PMR.

**Table 1:** Summary of 2018 recommendations for FDG-PET/CT(A) imaging in large vessel vasculitis and polymyalgia rheumatica by (a) the EANM, SNMMI, and the PET Interest Group (PIG), endorsed by the ASNC (10) and (b) a EULAR study group (11).

**Topic**	**EANM, SNMMI, and PET interest group**	**EULAR study group**
PET/CT	Low-Dose non-contrast CT for attenuation correction and anatomical reference	Hybrid PET with low-dose CT
Dietary preparation	Fasting for at least 6 h prior to FDG administration, intake of non-caloric beverages allowed. In case of FUO or suspected cardiac involvement: Consider fat-enriched diet lacking carbohydrates for 12–24, 12–18 h fast, and/or use of i.v. unfractionated heparin ~15 min prior to FDG injection	
Blood glucose level	Normal blood glucose levels desirable, but glucose levels <7 mmol/L (126 mg/dL) preferable	Preferably <7 mmol/L (126 mg/dL) Acceptable <10 mmol/L (180 mg/dL)
Glucocorticoids	Withdraw or delay GC therapy until after PET, unless there is risk of ischemic complications, as in case of GCA with temporal artery involvement. FDG-PET within 3 days after start of GC is optional as possible alternative	
Dose of FDG injection	3D: 2–3 MBq/kg (0.054–0.081 mCi/kg) body weight (depending on vendor suggestion of camera system)	
Interval between FDG administration and imaging	Minimum interval of 60 min between FDG administration and acquisition for adequate biodistribution	≥60 min, preferably 90 min
Position of patient	Supine, arms next to the body	Supine, arms should be down
Body parts to include	Head down to feet	From top to head to at least midthigh, preferably to below the knees
Scan duration	3D: 2–3 min/bed position (depending on vendor suggestion of camera system)	
Scoring FDG uptake	Use of standardized grading system proposed: 0 = no uptake (≤mediastinum); 1 = low-grade uptake (<liver); 2 = intermediate-grade uptake (= liver), 3 = high-grade uptake (>liver), with grade 2 considered possibly positive and grade 3 positive for active LVV. Typical FDG joint uptake patterns* should be reported if present. Normalization of arterial wall uptake to background activity of venous blood pool provides good reference for assessing vascular inflammation. Grading of arterial inflammation against liver background is established method	Qualitative visual grading; if result is unclear, compare it with liver background (grading 0–3)
Diagnostic performance for LVV and PMR	Based on available evidence, FDG-PET imaging exhibits high diagnostic performance CTA and FDG-PET have complementary roles in diagnosis of LVV	
Diagnostic performance for LVV and PMR	FDG-PET/CT(A) may be ofvalue for evaluating response to treatment by monitoring functional metabolic information and detecting structural vascular changes (evidence level III, grade C), but additional prospective FDG-PET/CT(A) studies are warranted	

CT, computerized tomography; CTA, CT angiography; FDG, ^18^F-Fluorodeoxyglucose; FUO, fever of unknown origin; GC, glucocorticoids; LVV, large vessel vasculitis; PET, positron emission tomography; PMR, polymysalgia rheumatic.

* Including scapular and pelvic girdles, interspinous regions of cervical and lumbar vertebrae, or knees.

At this time in 2018, FDG-PET/CT was still considered as not disease-specific and primarily developed to diagnose malignant and infectious/inflammatory diseases (10). The main limitation of FDG-PET/CT with or without angiography (A) to becoming a standardized diagnostic tool for large vessel arteritis was still the lack of an internationally accepted definition of vascular inflammation, based on the intensity and pattern of the glucose analog uptake (10).

According to the 2018 EULAR recommendation, PET was not recommended for the assessment of inflammation of cranial arteries, but may be used for detection of mural inflammation and/or luminal changes in extracranial arteries to support the diagnosis of large vessel GCA and as alternative imaging modality in patients with suspected TAK (11). A limitation to both of these recommendations is that in the clinical setting FDG-PET/CT imaging is certainly not routinely applied for diagnosing PMR because of its availability and the priority of sonography which was already introduced in the 2012 provisional classification criteria for PMR (12).

### Bone scintigraphy in current rheumatology

Since decades, bone scintigraphy with the radioactive tracer Technetium-99m pertechnetate (99mTc-pertechnetate) has been used for the diagnosis and follow-up of metabolic bone diseases, although diffuse scintigraphic changes are generally of little diagnostic value (13). Because of its high sensitivity and the easily acquired image of the whole body, its most common use was the detection of fractures in osteoporosis, pseudofractures in osteomalacia and the evaluation of Paget's disease (14).

Today, ^18^F-fluoride as tracer for PET/CT has shown to be more sensitive and specific than traditional bone scintigraphy, and the addition of CT further increases specificity in detecting bone metastasis (15). With 15–30 min vs. 3–4 h, uptake times are shorter than for conventional bone scintigraphy, and imaging times are shorter but the radiation exposure is approximately double with Fluoride PET/CT compared to standard bone scintigraphy (16). The main indications for ^18^F-fluoride PET/CT are bone metastases, only limited data are available for metabolic bone diseases.

Despite the limited value of bone scintigraphy in the primary diagnostic procedure, bone scintigraphy is of value in the preparation and follow up of radiosynoviorthesis. The method then allows direct comparisons before and after radiosynoviorthesis. Almost all national procedure guidelines include bone scintigraphy or other imaging techniques as mandatory before and 6 months after radiosynoviorthesis.

### Semiquantitative sialoscintigraphy and Sjögren's disease

According to the revised version of the European criteria proposed by the American-European Consensus Group for the classification of primary Sjögren's syndrome in 2002, a positive scintigraphy was defined as delayed uptake, reduced concentration, and/or delayed excretion of the radioactive tracer Technetium-99m pertechnetate (99mTc-pertechnetate) (17).

Already in 2007, it was foreseeable that the role of sialoscintigraphy will be reduced when applying the American-European criteria (AECG) (18). Since then, two separate arguments support this assumption: First, according to a meta-analysis with pooled sensitivity of 80% and specificity of 89%, the diagnostic accuracy of salivary sonography is comparable with sialography in patients with Sjögren's disease (19). Thus, the sonographic finding of major salivary gland involvement was proposed to replace sialoscintigraphy in the AECG criteria for diagnosis of primary Sjögren's syndrome (20). Second, due to the low specificity and the inability to differentiate uptake failure from secretory failure, specialists proposed that scintigraphic examination should focus on the degree of salivary gland dysfunction rather than the diagnosis or classification of primary Sjögren's syndrome (21). In 2016 then, new criteria were designed and validated by the ACR and EULAR, without using sialography at all (22).

Although in the cohort at the National Institutes of Health, USA, the older AECG set and the new 2016 ACR-EULAR set were found to be equivalent (23), sialoscintigraphy is currently not recommended for the classification of Sjögren's syndrome and may only remain a possible, but rarely indicated technique for the assessment of salivary gland dysfunction in clinical and research settings.

## Nuclear medicine techniques for rare rheumatological conditions

### Musculoskeletal indications

In rheumatology, nuclear medicine techniques are not recommended for the routine assessment of arthritis, enthesitis, dactylitis or spondyloarthritis. Only in rare cases, it may be exceptionally indicated.

#### Spondyloarthritis and rheumatoid arthritis

Scintigraphy of the sacroiliac joints is of limited value for the diagnosis of axial Spondyloarthritis, but unilateral compared to bilateral sacroiliitis is slightly superior despite low sensitivity (with sensitivities of scintigraphy for unilateral or bilateral, bilateral and isolated unilateral sacroiliitis of 64.9, 40.2, and 24.7%, and specificities of 50.5, 57.7, and 92.8%, respectively) (24). Therefore, scintigraphy is used only very rare in case of a contraindication for MRI.

Concerning FDG-PET in rheumatoid arthritis, a recent study finds even more harm than benefit for the patients (25). This unblinded study showed that FDG-PET/CT allowed incidental detection of extra-articular abnormalities in 57% of the patients, resulting in additional diagnostic procedures in 26.6% of them. Most important, 7.4% of the patients were suspected with a malignancy, but none turned out to be malignant—but as many as six clinical malignancies developed during follow-up, who were all negative on baseline FDG-PET/CT.

#### Adult-onset Still's disease

Several groups propose FDG-PET for the diagnosis and assessment of disease activity in adult-onset Still's disease (AOSD). Indeed, the glucose metabolism of liver, spleen and bone marrow were correlated with laboratory inflammatory markers, and FDG uptake in the spleen was proposed as a potential biomarker for predicting clinical prognosis of AOSD patients (26).

#### Polymyalgia rheumatica

According to the 2012 provisional EULAR/ACR classification criteria for PMR, sonography has been implemented as additional imaging option to support the classification (12). This consensus was based on data from a prospective cohort using clinical characteristics and laboratory data, together with sonography at least in several of the participating centers, but not FDG-PET.

A recent systematic review and meta-analysis now concludes that significant FDG uptake at a combination of anatomic sites is informative for diagnosis of PMR (27). According to a recent study significant FDG-uptake at least in three sites identified PMR with a sensitivity of 86% and a specificity of 85.5% in inflammatory rheumatic patients (28), whereas others showed that presence of optimal ordered combination of two sites already had a sensitivity and specificity to diagnose PMR of 73.2 and 87.5% in the training cohort and 78.6 and 80.1% in the validation cohort, respectively (29). When comparing these results with data from rheumatologists trained in sonography, both sensitivity (with 92.6%) and specificity (with 91.3%) were much higher than using FDG-PET (30). Thus, PMR can be helpful but is not considered as typical indication for FDG-PET.

Only in rare cases of “untypical” PMR, other diseases like large vessel arteritis, malignancies or infections may be detected using FDG-PET (31).

### Non-musculoskeletal indications

The most frequent non-musculoskeletal indications for FDG-PET are fever and elevated erythrocyte sedimentation rate of unknown origin. Besides, if nuclear medicine imaging has been applied for non-rheumatological indications it may occasionally provide additional information useful for the rheumatologist. Non-musculoskeletal indications include all assessments of organs involved in systemic rheumatic diseases, including the central nervous system (e.g., for apoplectic insult), the heart (e.g., for myocardial infarction) and the lungs (e.g., for pulmonary embolism).

#### Fever or elevated erythrocyte sedimentation rate of unknown origin

In 2017, from a systematic review with meta-analysis of the literature until 2015 together with a DELPHI exercise to evaluate the diagnostic yield of combined FDG-PET/CT in fever of unknown origin it was concluded, that the pooled diagnostic yield was 56%, with an estimated yield beyond conventional CT of 32%, —which was considered as insufficient evidence to support the value of FDG-PET/CT in investigative algorithms of fever of unknown origin (32).

In 2018 then, using FDG-PET in a cohort of 240 patients with fever or inflammation of unknown origin, diagnosis could be established in 79.2% of the patients (33). AOSD was diagnosed in 15.3% of patients with fever of unknown origin, large vessel vasculitis and PMR in 21.1 and 18.3% of the patients with inflammation of unknown origin, respectively, and IgG4-related disease in 15.4 of the patients after fever or inflammation of unknown origin.

#### Sarcoidosis

FDG-PET is usually not indicated for the assessment of musculoskeletal manifestations, as sonography and other imaging techniques are available. As FDG-PET, however, has a role for functional imaging in sarcoidosis, and positive pulmonary FDG-PET findings were shown to occur in two-thirds of patients with radiographic stage II and III sarcoidosis, occasional findings of arthritis may lead the patient to a rheumatologist. Negative pulmonary FDG-PET findings were common in patients with radiographic stage 0, I, and IV sarcoidosis, but do not exclude inflammatory findings in the musculoskeletal system (34).

#### Idiopathic retroperitoneal fibrosis

Diagnosis of idiopathic retroperitoneal fibrosis (iRPF) may be difficult, but important to be differentiated from malignant diseases. Therefore, FDG-PET was validated to distinguish between iRPF and malignancies, and indeed iRPF displayed a lower frequency of high-FDG-uptake retroperitoneal lesions and a lower mean maximum standardized FDG-uptake value (35). When combining the FDG-PET findings with the location of specific lymph nodes at axillary, retroperitoneal, supraclavicular, inguinal, or peritoneal sites, the area under the curve for the logistic regression model combining the lesions above renal arteries, a sensitivity of 90.5% and a specificity of 98.6% was reached for the maximum standardized uptake value. The authors concluded that FDG PET/CT can help to distinguish iRPF from retroperitoneal lymphoma and metastatic malignancy. During follow-up, persistent FDG uptake may help to better stratify the risk of relapse and target therapy (36).

## Conclusions

Nuclear medicine techniques are routinely used for the assessment of large vessel vasculitis like GCA and TAK, for diagnosis of rare metabolic bone diseases like Paget's disease and the assessment of salivary gland dysfunction in primary Sjögren's syndrome.

At present, FDG-PET is the nuclear medicine technique most often used in rheumatology. Combined with CT, MRI or angiography, it provides both functional and structural insights, which can be important for diagnostic or follow-up purposes. Also the use of FDG-PET in patients with fever or elevated erythrocyte sedimentation rate of unknown origin may lead to otherwise overseen rheumatologic diagnoses. Only rarely, FDG-PET is applied for diagnosis of adult Still's disease, sarcoidosis and idiopathic retroperitoneal fibrosis. Other nuclear medicine techniques may be more appropriate for assessment of specific organ involvements in systemic rheumatic diseases.

Indeed, there are several research gaps for use of nuclear medicine in rheumatology. First, sensitivity and specificity of different nuclear medicine techniques are not always well-established for each indication, especially for the rarest of the rheumatic diagnoses. Therefore, larger, controlled, and investigator-blinded studies are needed for further validation of existing tracers and development of more specific new markers.

## Author contributions

MW and MS contributed equally to the design, concept, and finishing of this article. All authors contributed to the article and approved the submitted version.

## Funding

The work was partly funded by the Verein zur Förderung der Hämatologie, Onkologie und Immunologie, Innsbruck, Austria.

## Conflict of interest

The authors declare that the research was conducted in the absence of any commercial or financial relationships that could be construed as a potential conflict of interest.

## Publisher's note

All claims expressed in this article are solely those of the authors and do not necessarily represent those of their affiliated organizations, or those of the publisher, the editors and the reviewers. Any product that may be evaluated in this article, or claim that may be made by its manufacturer, is not guaranteed or endorsed by the publisher.
